# Integrated transcriptomics and metabolomics analysis of flower petals color transition in different phenotype of *Lonicera macranthoides*

**DOI:** 10.3389/fpls.2025.1605238

**Published:** 2025-07-10

**Authors:** Jiayuan Zhu, Meiling Qu, Juan Zeng, Jiawei He, Jingyu Zhang, Simin Zhou, Qiaozhen Tong, Xiangdan Liu, Ribao Zhou

**Affiliations:** ^1^ School of Pharmacy, Hunan University of Chinese Medicine, Changsha, Hunan, China; ^2^ Department of Pharmacy, Key Laboratory of Germplasm Resources and Standardized Cultivation of Bulk Taoist Medicinal Herbs from Hunan, Changsha, Hunan, China; ^3^ Key Laboratory of Modernization of Chinese Medicine, Hunan General Higher Education Institutions, Changsha, Hunan, China; ^4^ Department of Pharmacy, Hunan Provincial Engineering Research Center for Standardization and Functionality of Chinese Medicinal Tablets, Changsha, Hunan, China; ^5^ Interdisciplinary and Intelligent Seed Industry Equipment Research, Yuelushan Laboratory, Changsha, China

**Keywords:** *Lonicera macranthoides*, wild-types, Xianglei-types, petal color, pigment

## Abstract

**Background:**

*Lonicera macranthoides* is a classic Chinese medicinal herb with direct flower color variation among types. Our group found differences in petal color transition between Xianglei-type (XL) and Wild-type (WT). At f1-f4 stage, the two types were green, and gradually changed from green to greenish white with development, in f5-f6, WT from white to golden yellow, but XL has little color change.

**Methods:**

Combined with transcriptomics and metabolomics analysis, the color conversion differences between XL and WT petals of *Lonicera macranthoides* were analyzed.

**Results:**

Significant differential genes were identified at f1-f4, f5 and f6 in WT and XL, 14528, 7955 and 17985, respectively. At f1, the WT anthocyanin gene showed lower than XL (*P* < 0.05), significantly down-regulated XL and up-regulated WT at f2 (*P* < 0.05), but still showed higher XL than WT. XL showed significantly lower *CHS* (DN46824_c0_g4), *CHI* (DN43583_c0_g1), ANS (DN28844_c0_g1) than WT in f3. f4 stage, but again XL anthocyanins were higher than WT. We found that XL carotenoid genes all showed significantly higher levels than WT in f1 (*P* < 0.05). XL were significantly down-regulated at f2-f3 (*P* < 0.05), but not WT. Surprisingly, WT had a rapid rise in *PDS* (DN55130_c2_g1), *ZDS* (DN54194_c0_g1), and *BCH* (DN42921_c1_g2) at f4, far exceeding XL (*P* < 0.05). *PSY*, *PDS*, and *ZDS* genes on the carotenoid synthesis pathway, and *CHS* and *CHI* genes on the anthocyanin synthesis pathway were identified to have lower XL than WT at f5. The anthocyanin synthesis pathway *CHS*, *CHI*, and *ANS* were more expressed in XL than WT at f6, whereas the carotenoid synthesis pathway *BCH*, *LCYB*, and *NXS* were more expressed in WT than in XL. Expression validation of these genes was performed using quantitative real-time PCR (qRT-PCR). Metabolomic analysis identified a total of 158 flavonoids and one carotenoid. There were few pigment-related metabolites of f1-f4, WT had higher β-carotene content in f5 than XL, Pelargonin, marasin-3-O-galactoside had the most content in XL, and cyanidin had the most content in WT of f6. Weighted gene co-expression network analysis (WGCNA) showed that two gene modules and one gene module were strongly associated with anthocyanin and β-carotene synthesis, respectively. Genes associated with carotenoid synthesis in the modules identified by KEGG annotation were *PSY*, *PDS*, *Z-ISO*, *ZDS*, *LCYB*, *BCH*, and *NXS*.

**Conclusion:**

Our results provide an overall understanding of the regulatory mechanisms underlying differences in petals color transition of different phenotypes of *Lonicera macranthoides*.

## Introduction

*Lonicera macranthoides* Hand-Mazz is one of the traditional Chinese medicinal herbs the family Caprifoliaceae. Fresh flowering or dried flower buds were utilized as medicine (Qiu et al., 2023). The health advantages of honeysuckle were initially recorded by “The Tang Materia Medica”. The plant has a chilly, sweet flavor and is connected to the meridians of the stomach, heart, and lungs, according to TCM philosophy. Numerous illnesses can be treated with it, but the most prevalent ones are skin and inflammatory conditions (Lee et al., 2021; Li et al., 2021). Researchers created excellent asexual varieties called “*Lonicera macranthoides*” because the WT *Lonicera macranthoides* has the drawbacks of irregular flower buds, short flower bud stage, and immediate withering 1~2 d after corolla expansion. This makes it easy to cause resource waste and a decrease in the quality of medicinal materials due to premature harvest. Its more than 20 d flowering duration significantly lessens the loss. According to previous sample analysis by our group, the WT petal color changed from yellowish white to golden yellow between f5 and f6, but the XL color change between f5 and f6 was not very different (Fu et al., 2024), so it was speculated whether petals color transition difference was caused by the difference in the accumulation of pigment-related metabolites between the two types.

The following three classes of substances-flavonoids, betaines, and carotenoids-are secondary metabolites that influence how plants develop their color; the majority of plants include both types of molecules (Qiu et al., 2023). The six most common anthocyanins in flowers are mulberry pigments, petunidin, delphinidin, paeonidin, geranidin and mallow pigments (Zhao et al., 2022). Anthocyanins, as important constituents of flavonoids, are predominantly yellowish, reddish, violet and violet to blue in petals (Qiu et al., 2023; Vidana Gamage et al., 2021). Lutein and β-carotene are the major carotenoids that give the petals a pale to dark yellow color (Nisar et al., 2015). Taken together, flavonoids and carotenoids co-regulate flower color changes.

Flavonoid and carotenoid biosynthesis pathways have been well characterized in many plants, for example, *Arabidopsis* (Broun, 2005), *Dioscorea alata* L (Wu et al., 2015), and *Paeonia delavayi* (Zou et al., 2023). Anthocyanins, as a subgroup of flavonoids, present color to plants by modifying anthocyanins with sugars and acylic acids. Anthocyanin biosynthesis is mainly related to the phenylalanine metabolic pathway, and the key enzymes involved in the early stage of anthocyanin synthesis are chalcone isomerase (*CHI*), chalcone synthase (*CHS*), flavonoid 3′-hydroxylase, and flavanone 3-hydroxylase (*F3H*) (Sánchez-Cabrera et al., 2021); while those in the later stage are anthocyanin 3-glycosyltransferase (F3′ H), anthocyanin synthase (*ANS*), and dihydroflavonol 4-reductase (*DFR*) (Tohge et al., 2017; Wang et al., 2017). Phytoene synthase (*PSY*), phytoene desaturase (*PDS*), 15-cis-ζ-carotene isomerase (*ZISO*), ζ-carotene desaturase (*ZDS*) and carotenoid isomerase (*CRTISO*) can be sequentially converted to red lycopene, which can be further differentiated into yellow β-carotene and α-carotene by lycopene β-cyclase (*LCYB*) and lycopene ϵ-cyclase (*LCYE*), respectively, as precursors of carotenoids (Yuan et al., 2015). Afterwards, Carotene ϵ-monooxygenase (*CYP9*) catalyzes α-carotene to form xanthophylls with antioxidant effects. B-Carotene can be converted by carotenoid β-hydroxylase (*BCH*) to generate yellow zeaxanthin, an isomer of lutein (Li Y. et al., 2022; Sun et al., 2022).

Transcriptomics and Metabolomics can be used to study the mechanisms underlying plant growth and development. UHPLC-MS was used in this investigation to identify the metabolites of f5 and f6 of WT and XL *Lonicera macranthoides*. Differentially expressed metabolites (DEMs) and differentially expressed genes (DEGs) were examined utilizing transcriptomics and metabolomics. Correlation analysis of the information gathered using these techniques confirmed the production of carotenoids and anthocyanins. In order to clarify the molecular foundation and metabolic mechanisms underlying the variations in petals color transition differences between XL and WT, this work links the existence of DEGs and DEMs in anthocyanin and carotenoid production pathways.

## Materials and methods

### Plant material

On June 27, 2022, our research team collected samples of the XL and WT of *Lonicera macranthoides* from f5 and f6 in Longhui County, Shaoyang City, Hunan Province (27° 7 ′ N, 111° 1 ′ E). Professor Ribao Zhou of Hunan University of Chinese Medicine identified and separated the samples into two varieties, XL and WT. Our group identified and divided the two varieties into seven developmental stages (Fu et al., 2024; Liu et al., 2019; Zeng et al., 2025), and found that the petals color transition were in f1-f6, WT color changed from yellowish white to golden yellow, and XL changed from yellowish green to yellowish yellow. Therefore, f5 and f6 were selected as primary samples for transcriptomics and metabolomics analysis in this study. Six or more individual samples were then collected, three independent biological replicates for RNA library construction, three additional biological replicates for UHPLC-MS/MS analysis, and the remaining samples were frozen in liquid nitrogen and kept in a freezer at -80°C for further analysis (Li Y. et al., 2022).

### UHPLC-MS/MS analysis

Metabolite extraction and analysis were performed by Shanghai BIOTREE Biotechnology Co., Ltd. (Shanghai, China). Weigh 10 mg of each lyophilized sample and crush the sample using a blending mill (60 Hz for 30 seconds). The aforementioned samples were added to an Eppendorf tube after 500 μL of extraction solution (methanol/water = 3:1, precooled to -40°C, containing internal standard) was added. The extract was vortexed for 30 seconds, homogenized for 4 mins at 40 Hz, and then sonicated for 5 minutes in an ice-water bath. They were then centrifuged for 15 minutes at 12,000 rpm and 4°C after being kept on a shaker overnight (Hou et al., 2023). The resultant supernatant was diluted ten times with a methanol/water mixture (v: v = 3:1, containing internal standard), filtered through a 0.22 μm microporous membrane (Shanghai Jiecheng Biotechnology Co., Ltd.; batch number: 2810567236; size: 13 mm 0.22 μm), UHPLC separation was conducted via an EXIONLC system (Sciex). Two microliters of the sample was injected onto a Waters Acquity UPLC HSS T3 column (1.8 μm 2.1 × 100 mm) with the column temperature set at 40°C and a flow rate of 400 μL/min (Hou et al., 2023). Mobile term: 0.1% formic acid in water as phase a and acetonitrile as phase B. Separation is achieved according to the following gradient: Mass spectrometry was performed using a SCIEX 6500 QTRAP + Triple Quadrupole Mass Spectrometer (TQMS) equipped with an IonDrive Turbo VESI ion source in multiple reaction monitoring (MRM) mode. The ion source parameters were as follows: IonSpray Voltage: + 5500/-4500 V, Curtain Gas: 35 psi, Temperature: 400°C, Ion Source Gas 1:60 psi, Ion Source Gas 2:60 psi, DP: ± 100 V (Li et al., 2020).

### RNA extraction, cDNA library construction and sequencing, transcript level quantification, DEG, DEM screening

Total RNA was extracted using the Polyphenol Plant Total RNA Extraction Kit (Biospin, China, lot no. BSC65S1).The concentration and purity of RNA were then assessed using the Bioanalyser 2100 (Agilent, CA, USA) and RNA 1000 Nano LabChip kit (Agilent, USA, lot no. DP441). To produce cDNA libraries, cleaved RNA fragments were reverse transcribed using the mRNASeq sample preparation kit (Illumina, USA, lot no. NR605-C4). Using the Illumina Ltd. NovaseqTM 6000 platform, BioBioMedical Technology Co. (Shanghai, China) carried out paired-end sequencing on three biological replicates of each species. Low quality and poly-N reads were eliminated after raw data collection in order to assess sequence quality. Cutadapt was used to remove poly-A/T, sequences less than 100 bp in length after truncation, and reads with N content above 5%, sequencing adapters, low-quality bases, and undetermined bases. Sequence quality was then verified using FastQC (http://www.bioinformatics.babraham.ac.uk/projects/fastqc/).

(Cai et al., 2022). StringTiev1.3.4 (https://ccb.jhu.edu/software/stringtie/index.shtml?t=example) used to map the quality-filtered reads to the reference genome or transcriptome. After assembly and annotation, transcripts per million (TPM) values were calculated by SaLmon (version 0.8.2) to quantify gene expression. The DESeq program in R software was used to identify DEGs(both upregulated and downregulated genes), with |log2(fold-change)|> 2 or and *P* < 0.05 serving as the threshold for differential gene screening (Pertea et al., 2016). Compound Discoverer 3.1 was utilized to quantify the raw data for every metabolite. The mzCloud, mzVault, and Masslist databases were used to reasonably accurately characterize the final metabolites after peak extraction and peak area quantification. Molecular formulas were then inferred using peaks from molecular and fragment ions. The OPLS model prediction (VIP) score was used to screen DEMs (Zhou et al., 2022).

### Bioinformatics analysis of omics data

Pearson correlation analysis and PCA are the simplest methods to analyze the multivariate statistical distribution of features. In general, both within-group and between-group sample distributions can be presented. Pearson correlation analysis was performed using the “stats” package in R (version 3.5.0). Principal component analysis was performed by online OE clouds (Chia et al., 2020). Volcano-map can understand the number of up- and down-regulated DEGs and DEMs. KEGG enrichment analysis can know the pathways that are mainly enriched in significant difference DEGs and DEMs. Volcano-map was performed by the online OmicShare tool (Chen et al., 2015). KEGG enrichment analysis was performed for DEGs and DEMs, respectively. Structural genes of carotenoid and anthocyanin production pathways and associated DEMs were subsequently selected from DEGs, respectively, and heatmaps of gene expression and metabolite content were created by R v4.1.2 (Qiu et al., 2023).

To select genes potentially involved in anthocyanin and carotenoid associations, genes were identified via the Sangerbox 3.0 public website (http://sangerbox.com/home.html), weighted gene co-expression network analysis (WGCNA) was performed on f5 and f6 critical period DEGs. Weighted adjacency matrices among different genes were constructed with power functions, and a dynamic tree cutting procedure (combined cut height < 0.25, minimum module size 30) was used to screen for similar modules in graded trees. Modular signature genes are defined as the first principal component of a given module and are used to represent the expression profiles of module genes in each sample. Pearson correlation analysis between each module signature gene and anthocyanins and carotenoids was performed using this website.

### qRT-PCR validation

Candidate gene sequences were searched using a transcriptomic database and NCBI Primer-Blast created specific primers used in qRT-PCR (**Supplementary Table S1**) (Fang et al., 2020).Total RNA from the f5 and f6 WT and XL of Lonicera macranthoides was extracted using the Biospin polysaccharide phenol kit (Bozhi Technology Co., Ltd., Hangzhou, China, lot number: BSC65S1).Total RNA was then isolated from Stage 5 and Stage 6 of L. griseus from WT and XL breeds using PCR created by RevertAid First Strand cDNA Synthesis (Thermo Fisher Scientific, USA, Lot No. CW0682). cDNA was generated by RNA reverse transcription (Yu et al., 2021). qRT-PCR was performed to analyze gene expression levels, three biological replicates and three technical replicates were performed, and the reliability of the data was verified.18s RNA was used as an internal reference gene. Statistical analysis was performed using SPSS 21.00 software, and data collected in each group were expressed as mean standard deviations. Statistically significant differences were present if *P* < 0.05 and highly significant if *P* < 0.01; otherwise, statistical significance was absent.

## Results

### Morphology analysis of *Lonicera macranthoides*

**Figure 1:**
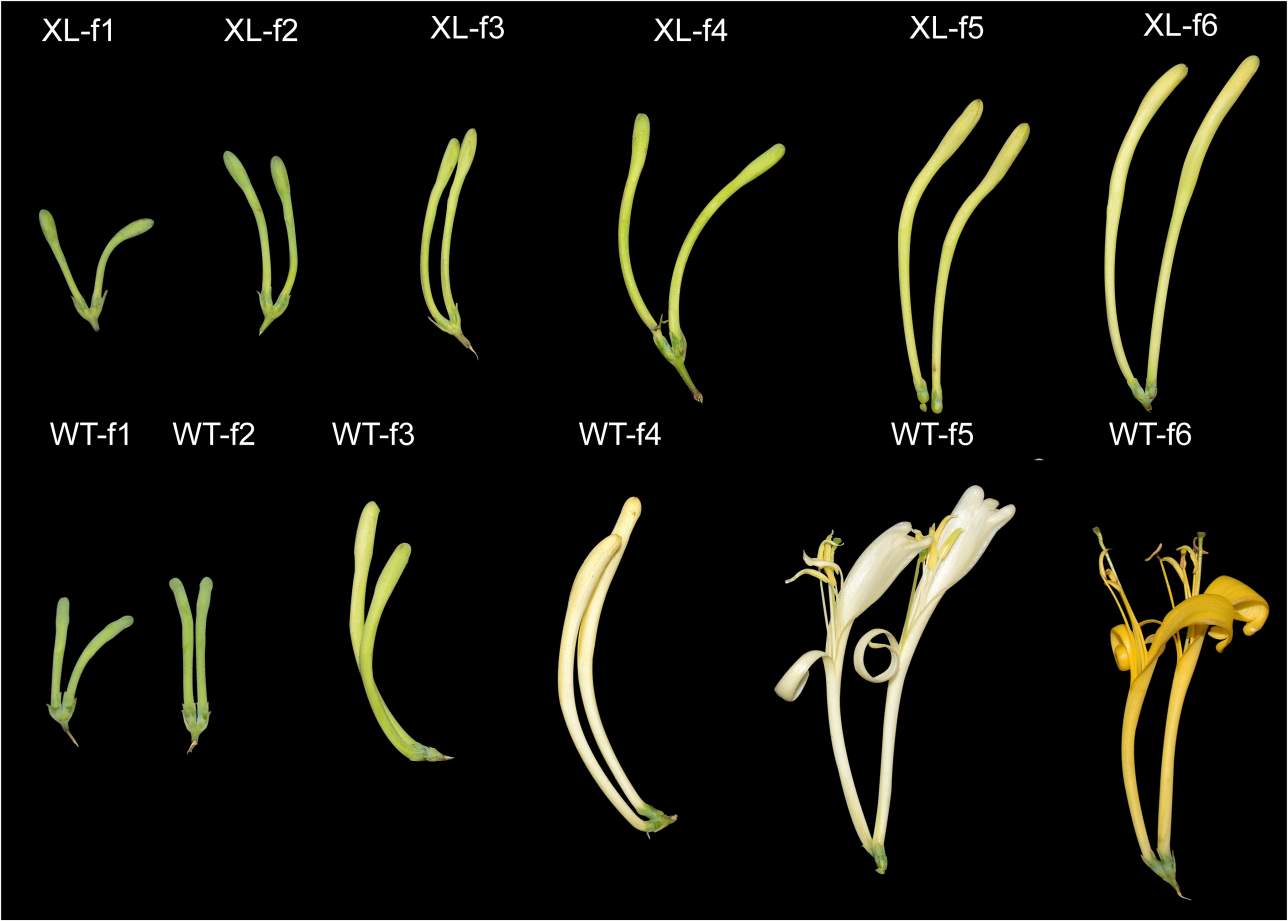
Phenotypic changes of *Lonicera macranthoides* f1-f6 in WT varieties and XL varieties. XL varieties do not bloom, WT varieties become cone-shaped florets, flower stage 5 for the full bloom stage (f5), buds length is about 1.5 cm. Six developmental stages of WT were described: WT-f1: early bud stage, WT-f2: green bud stage, WT-f3: green-white bud stage, WT-f4: white bud stage, WT-f5: white flower stage, and WT-f6: golden yellow flower stage. Six developmental stages of XL are described: XL-f1: early flower bud stage, XL-f2: green flower bud stage, XL-f3: light green flower bud stage, XL-f4: green and white flower bud stage, XL-f5: white flower bud stage, and XL-f6: yellow and white flower bud stage.

WT and XL were green and green-white at stages f1-f4. During the development of *Lonicera macranthoides*, both WT and XL flower petals color transition at f5 and f6, WT opened at f5 and petals color changed from white to golden yellow, XL did not open, and petals almost unchanged in color (**Figure 1**).

### Analysis of DEGs during f1-f6

**Figure 2:**
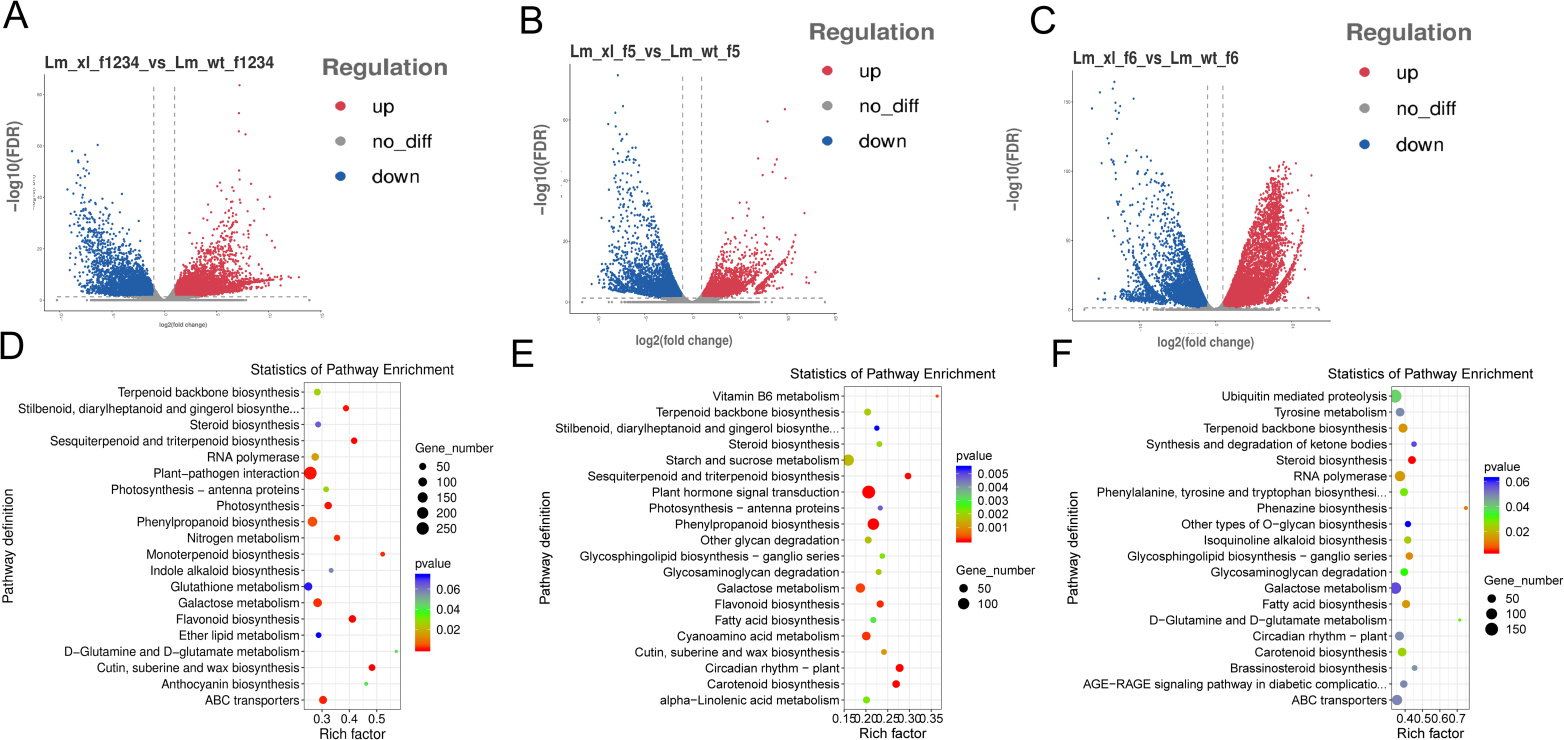
DEGs Characterization between f1-f6 Samples the WT and XL varieties of *Lonicera macranthoides*. **(A)** volcano plot of DEGs identified at f1-f4. **(B)** volcano plot of DEGs identified at f5. **(C)** volcano plot of DEGs identified at f6. **(D)** KEGG enrichment analysis of DEG at f1-f4. **(E)** KEGG enrichment analysis of DEG at f5. **(F)** KEGG enrichment analysis of DEG at f6. In KEGG enrichment maps, circle size represents the number of genes, and color responds to *P*-value.

In order to understand more about the composition of anthocyanins in WT and XL of *Lonicera macranthoides*, the transcriptomics of corolla of f1-f6 at two developmental phases were compared pairwise to find DEGs (Deng et al., 2023). According to PCA correlation investigations, the transcriptomics data of *Lonicera macranthoides* were demonstrated to be reproducible under the same variety, but different in other varieties. These findings suggest that transcriptomics data are reliable and suitable for in-depth examination analyses (**Supplementary Figure S1**). We discovered 14528 DEGs in WT-f1234 *vs* XL-f1234,7955 in WT-f5 *vs*. XL-f5 and 17985 in WT-f6 *vs*. XL-f6 (**Supplementary Tables S2**-**S4**). WT-1234 *vs* XL-1234, 8856 genes were upregulated and 5672 genes were downregulated. WT-f5 *vs* XL-f5, 3894 genes were upregulated and 4101 genes were downregulated. WT-f6 *vs* XL-f6, 10,065 genes were upregulated and 7,920 genes were downregulated (**Figures 2A-C**). In addition, according to KEGG pathway enrichment analysis, DEGs of f1-f4, f5 and f6 contained 18 different metabolic pathways and two biological systems. f1-f4 was significantly enriched in flavonoids, anthocyanins, and ABC transporters (transport anthocyanin-related proteins), f5 was quite enriched in the photosynthesis-antennase, flavonoid, and carotenoid biosynthetic pathways, but f-6 was more concentrated in ABC transporters and carotenoids (**Figures 2E, F**). The above findings clearly indicate that the differences in corolla color transition between XL and WT may be caused by flavonoid biosynthesis, carotenoid biosynthesis, and ABC transporters.

### DEGs involved in anthocyanin biosynthesis pathway and carotenoid biosynthesis pathway

**Figure 3:**
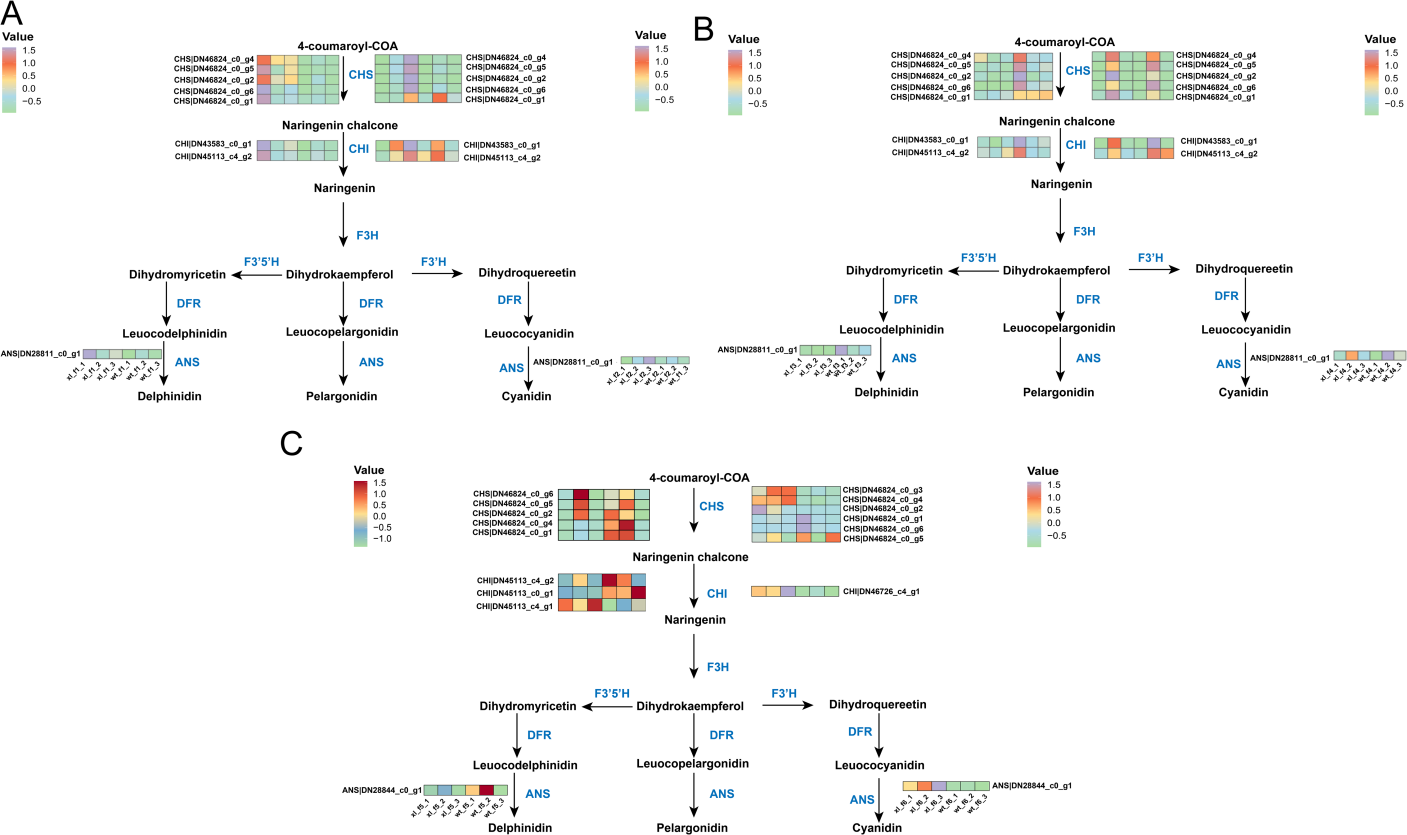
Overview of the anthocyanin biosynthetic pathway and the role of DEGs in XL and WT of *Lonicera macranthoides.* The data are the mean TPM value of three biological replicates. *CHS*, chalcone synthase; *CHI*, chalcone isomerase; *F3H*, flavone 3-hydroxylase; *F3’H*, flavone 3’-hydroxylase; *F3’5’H*, flavone 3’5’-hydroxylase; DFR, dihydroflavonol reductase; *ANS*, anthocyanidin synthase. In scale bar, the expression of DEGs is indicated in purple (high abundance) and green (low abundance). **(A)** f1 and f2; **(B)** f3 and f4; **(C)** f5 and f6.

The pathway of anthocyanin synthesis was shown, and the expression levels of the structural genes found were analyzed (Wu et al., 2023). Comparing XL and WT, we found that at f1, all identified enzymes involved in anthocyanin synthesis showed lower XL than WT (*P* < 0.05). At f2, all XL anthocyanin-related enzymes were significantly down-regulated, and WT was up-regulated (*P* < 0.05), but still showed higher XL than WT. At f3, the enzymes related to XL anthocyanin synthesis continued to decrease, in which *CHS* (DN46824c0g4), *CHI* (DN43583c0g1), and *ANS* (DN28844c0g1) were significantly lower than WT. At f4, however, anthocyanin-synthesis-related enzymes were down-regulated in both cultivars, but XL was again higher than WT (**Figures 3A, B**). We discovered that *CHS* (DN46824_c0_g6, DN46824_c0_g5) expression of XL was considerably lower at f5 (*P* < 0.05). These genes might have an impact on the structural differences between the two flowering colors at f5. There were other *CHS* (DN46824_c0_g2, DN46824_c0_g4, DN46824_c0_g1), but the two kinds did not differ significantly (*P* > 0.05). While the expression levels of other genes expressing *CHI* (DN45113_c0_g1, DN45113_c4_g1) did not change significantly, *CHI* (DN45113_c4_g2) was considerably reduced in XL (*P* < 0.05). Interestingly, despite being a crucial gene downstream of the anthocyanin biosynthesis pathway, the expression level of the *ANS* (DN28844_c0_g1) did not alter significantly between the WT and XL. The *CHS* (DN46824_c0_g3, DN46824_c0_g4, and DN46824_c0_g2) were substantially more abundant in XL at f6. On the other hand, WT had considerably lower expression levels of the genes encoding *ANS* (DN28844_c0_g1) and *CHI* (DN46726_c4_g1) than XL (*P*<0.05) (**Figure 3C**).

**Figure 4:**
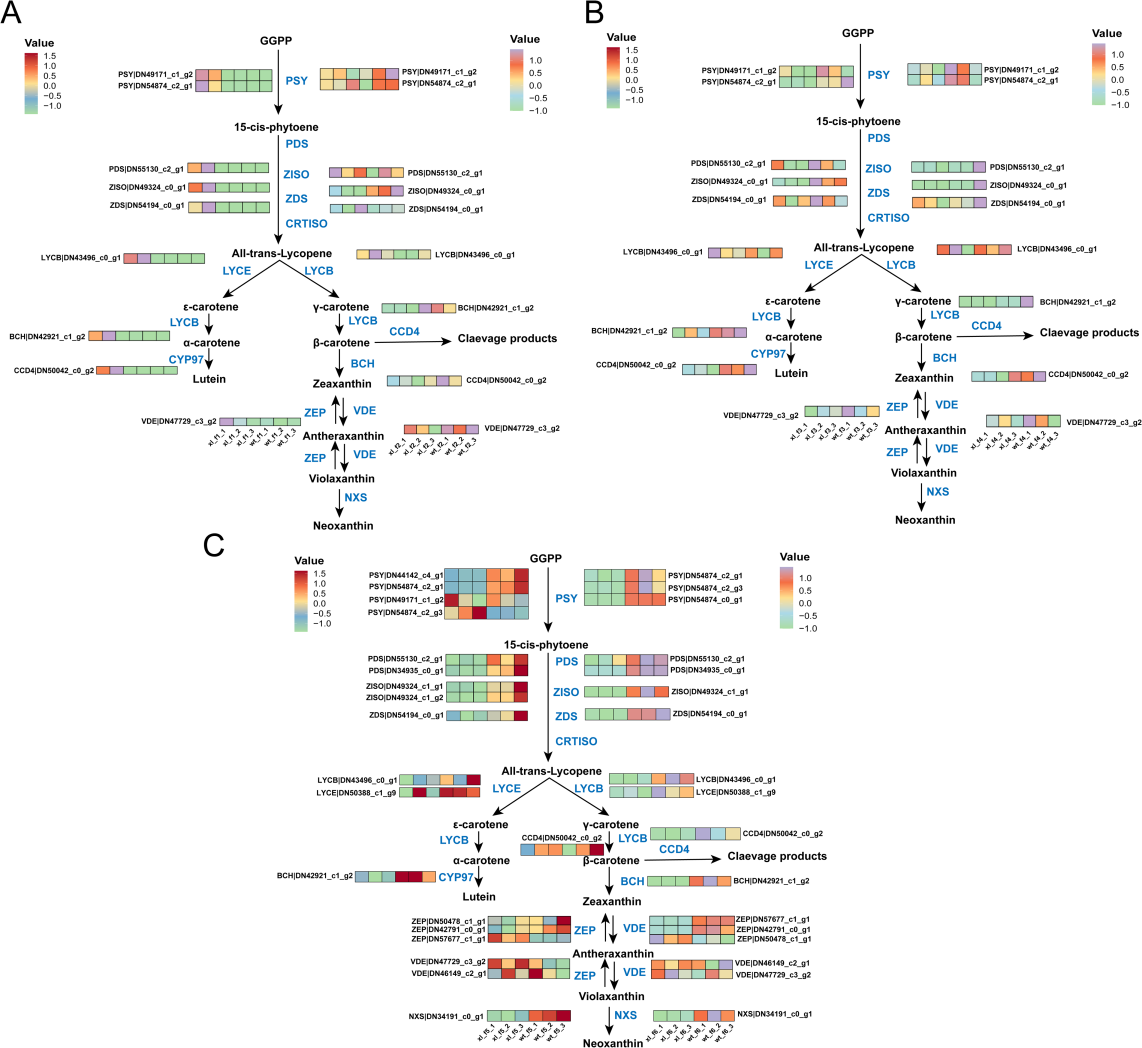
Overview of the carotenoid biosynthesis pathway and the role of DEGs in WT and XL varieties of *Lonicera macranthoides.* The data are the mean TPM value of three biological replicates. *PSY*, Phytoene syn-thase; *PDS*, phytoene desaturase*; Z-ISO*, zeta-carotene isomerase*; ZDS*, zeta-carotene desaturase*; LCYB*, lycopene beta-cyclase; *LCYE*, lycopene epsilon-cyclase; *CYP97*, cytochrome P450–type hy-droxylase; *BCH*, beta-carotene hydroxylase; *ZEP*, zeaxanthin epoxidase*; VDE*, violaxanthin de-epox-idase; *NXS*, neoxanthin synthase*; CCD4*, carotenoid synthase. In scale bar, the expression of DEGs is indicated in purple (high abundance) and green (low abundance). **(A)** f1 and f2; **(B)** f3 and f4; **(C)** f5 and f6.

Following mapping the metabolic pathway of carotenoid synthesis and analyzing the carotenoid-related DEGs, at f1 we found that XL carotenoid-related synthase genes all showed significantly higher than WT (*P* < 0.05). At f2, XL carotenoid-related enzymes were significantly down-regulated (*P* < 0.05), but WT was not significantly down-regulated, with *BCH* (DN42921_c1_g2) expressed significantly higher in WT than XL (P < 0.05). The f3 was generally consistent with the f2 trend and did not change significantly. Surprisingly, at stage f4, WT had cliff growth of *PDS* (DN55130_c2_g1), *ZDS* (DN54194_c0_g1), and *BCH* (DN42921_c1_g2), far exceeding XL (*P* < 0.05) (**Figures 4A, B**). We discovered that in f5, the genes encoding key genes upstream of the carotenoid synthesis pathway, such as *PSY* (DN44142_c4_g1, DN54874_c2_g1, and DN49171_c1_g2), *PDS* (DN55130_c2_g1, and DN34935_c0_g1), and *ZDS* (DN54194_c0_g1), were all significantly lower expressed in XL varieties than in WT varieties (*P*< 0.05). Zeaxanthin was synthesized and β-carotene was cleaved as a result of the expression of the genes encoding *BCH* and *CCD4*, respectively. In f6, the *BCH* (DN42921_c1_g2) was much less expressed in the XL than in the WT. Additionally, the XL DEG levels *LCYB* (DN43496_c0_g1) and *NXS* (DN34191_c0_g1) were significantly lower (*P*<0.05) than the WT, which was consistent with the finding that the XL corollas presented a yellowish-green, whilst the WT corollas showed golden yellow. Finally, it is anticipated that the WT displays golden yellow at f6, while the XL continues to display yellowish green; the XL is thought to display yellowish green at f5, while the WT displays yellowish white (**Figure 4C**). The different petal color of the two types may be caused by differences in expression of carotenoid and anthocyanin-related genes.

### Analysis of metabolites during f1-f6

UHPLC-MS was used to identify the metabolites of WT and XL of f1-f6 in order to obtain insight into the causes of the variation in flowering color between the two species of *Lonicera macranthoides* (Li J. et al., 2022). The instrument was stable, as evidenced by the good agreement between the TIC profiles of the QC sample peak sizes and retention periods, as determined by the overlay analysis of the total ion current (**Supplementary Figure S2A**). PCA showed that whereas biological replicates were closely clustered in the same breeds, “metabolite profiles” were expressed at different levels in the two breeds (**Supplementary Figure S2B**). These results imply that metabolomics data are repeatable and trustworthy.

**Figure 5:**
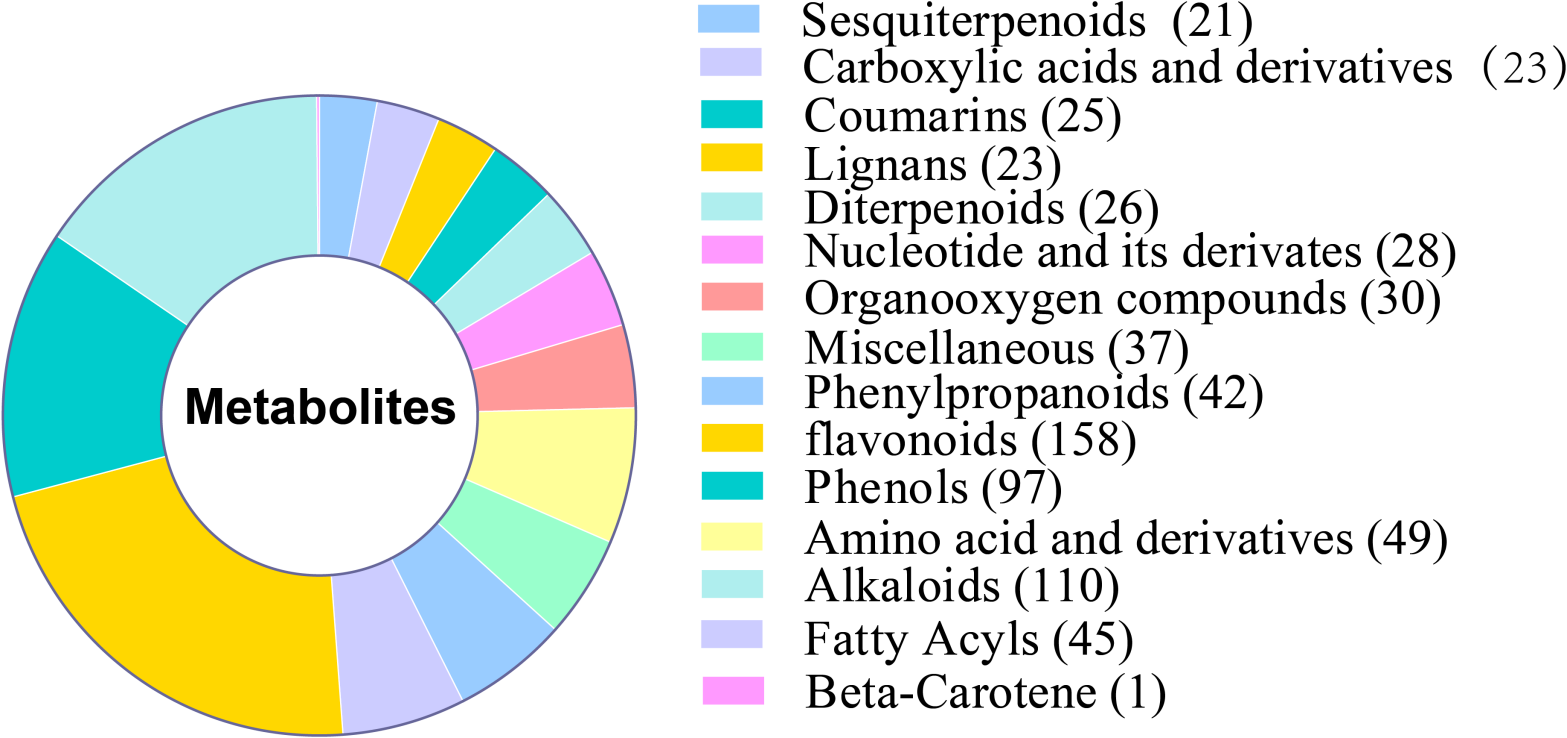
Analysis of identified metabolites of two types of *Lonicera macranthoides*.

Plant color is greatly influenced by anthocyanins, which are flavonoids. Depending on the B-ring substituent, plants have six primary types of anthocyanins: Mulberry pigments include delphinidin, petunidin, malvidin, pelargonin, cyanidin, and peonidin (Wu et al., 2023). 158 flavonoids, including 7 anthocyanins, were found in samples of the WT and XL types used in this study (**Figure 5**). Mulberry pigments, mallowins, geranins, and anthocyanins are the four categories of anthocyanins that have been identified. Lutein (45%), β-carotene (25%-30%), α-carotene, purple xanthophylls (10%-15%), and neoxanthophylls (10%-15%) are the most common plant carotenoids. The majority of plant species have similar carotenoid compositions, and plant carotenoids are isoprenoids (Morelli and Rodriguez-Concepcion, 2023; Nisar et al., 2015). The only carotenoid identified in this study was β-carotene. Additional anthocyanins were also found, including saffron yellow pigments and photopigments.

Two hundred and 36 flavonoid compounds were detected in f-1234 containing only one anthocyanin as Procyanidin B2, which showed significantly higher XL than WT, indicating less pigment accumulation in the prophase. 158 flavonoid compounds and 1 carotene compound detected in WT and XL were analyzed for their content at the f5 and f6 stages using HPLC-MS, and the accumulation of most anthocyanins and one carotene, including siilibinin, procyanidin B2, procyanidin B1, malvidin 3-O-glucoside, Cyanidin, Pelargonidin, Delphinidin, Gossyin, and Beta-Carotene, differed at the flower color transition stage between the two varieties (**Supplementary Tables S5**, **S6**).

### Analysis of DEMs during f1-f6

**Figure 6:**
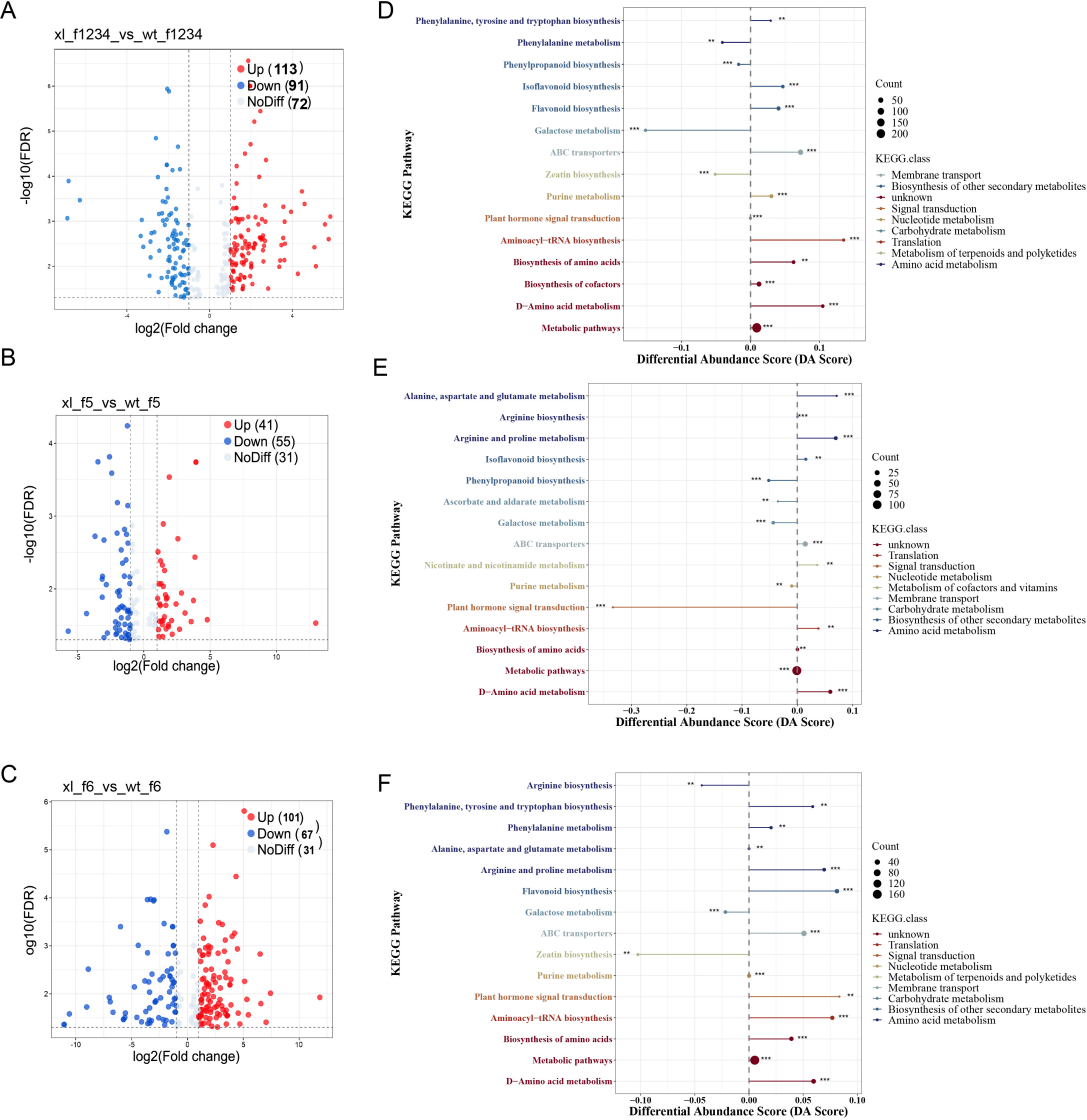
DEM analyses of f1-f6 of the two varieties. **(A)** volcano plot of differential metabolites at f1-f4. **(B)** volcano plot of differential metabolites at f5. **(C)** volcano plot of differential metabolites at f6. **(D)** KEGG enrichment plot of differential metabolites at f1-f4. **(E)** KEGG enrichment plot of differential metabolites at f5. **(F)** KEGG enrichment plot of differential metabolites at f6. The size of the point at the end of the line segment represents the number of DAVs involved in the pathway, while the length of the line segment indicates the absolute value of the DA score. For the points displayed in the right panel, the total expression of the pathway tends to be up-regulated the longer the line segment, * indicates the *p*-value.

We selected DEM based on PCA and OPLS-DA findings, FD and VIP (variable importance in prediction) statistics, and other data in order to more accurately identify metabolite accumulation patterns. f-1234 identified 204 DEMs, 113 upregulated, and 91 downregulated (**Figure 6A**). 41 and 55 of 96 DEMs found in f5 were upregulated and downregulated, respectively (**Figure 6B**). in f6, 168 DEMs were identified, 101 of which were upregulated and 67 downregulated (**Figure 6C**) (**Supplementary Tables S7**-**S9**). Subsequently, differential metabolites from each group were enriched, annotated, and divided into unique KEGG pathways. According to KEGG enrichment analysis, the significant enrichment at f1-f4 stage was the synthesis of flavonoids, and the differential metabolites of f5 and f6 significantly enriched the biosynthesis of carotenoids and anthocyanins, followed by the biosynthesis of isoflavones and ABC transporters. The results showed that f5 and f6 were the main stages of petals color change difference (**Figures 6D, E**).

### Metabolite analysis of main stages of petal color transitions difference

**Figure 7:**
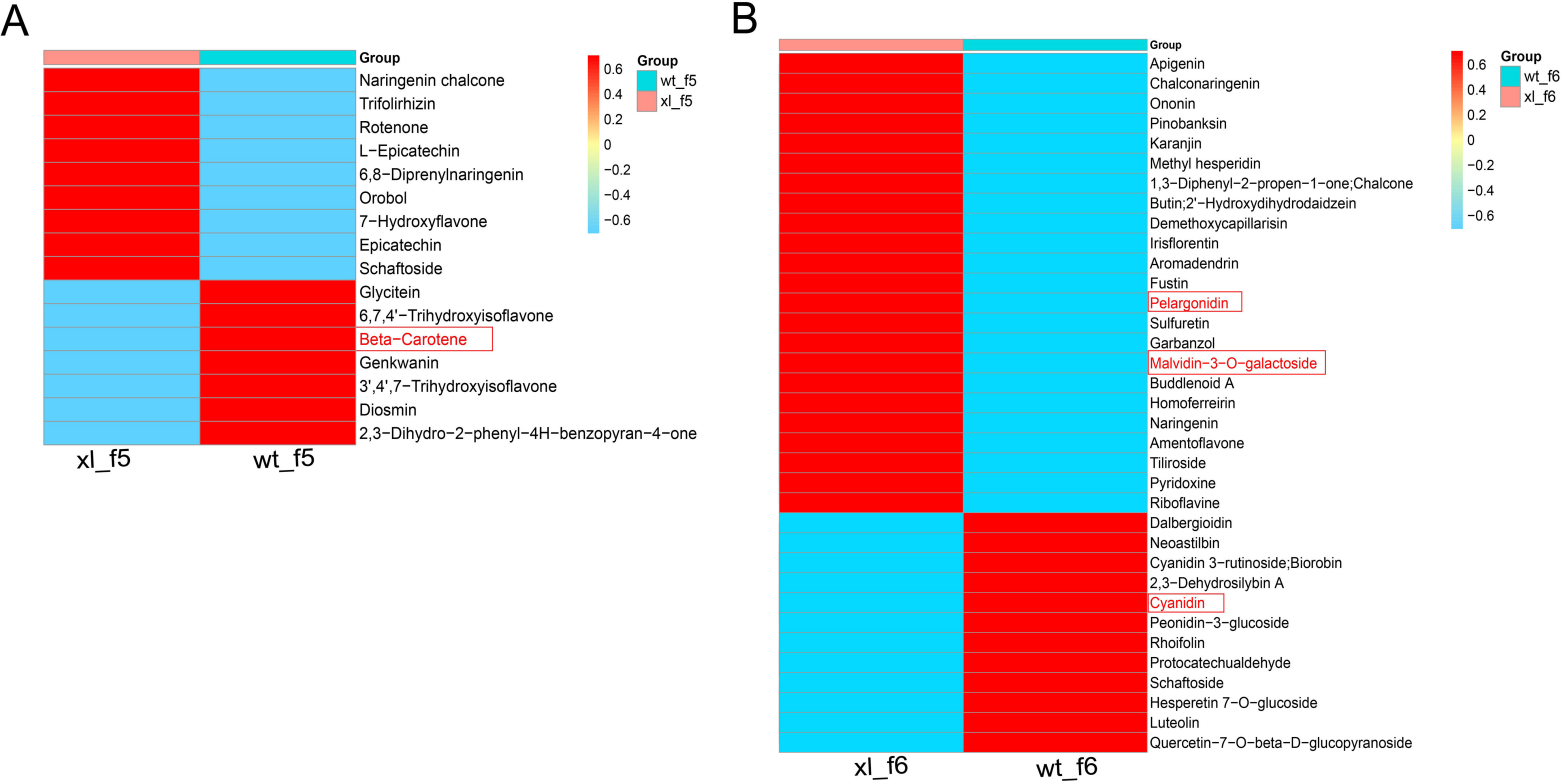
Heat map of differential metabolites involved in flavonoid and carotenoid biosynthesis pathways. **(A)** Heat map of f5 involved in flavonoid and carotenoid biosynthesis. **(B)** Heat map of f6 involved in flavonoid and carotenoid biosynthesis. The data are the mean contained value of three biological replicates. In scale bar, the expression of DEM is indicated in red (high abundance) and blue (low abundance).

A total of 15 flavonoids and 1 carotenoid were identified and quantified using a thermogram to assess the accumulation of metabolites linked to flavonoid synthesis during XL and WT of f5 (**Figure 7A**) (Li et al., 2022; Zhou et al., 2022). The WT included six distinct types of flavonoids, the XL contained nine different types, and the WT contained one carotenoid. XL had the highest levels of epicatechin, charophyllin, flavanone, and rotenone, while WT had the highest levels of corianderin, luteolin, naringenin, chalcone, and β-carotene. A total of 35 flavonoids were identified and measured in f6. There were twelve flavonoid in WT and twenty-three in XL (**Figure 7B**). Three distinct anthocyanin metabolites were detected at f6. XL had the largest levels of mallow pigment-3-O-galactoside and pelaggonin, whereas WT had the highest levels of cyanidin. These results showed that the production of pigments from *Lonicera macranthoides* was controlled by anthocyanins and carotenoids. WT exhibited increased gene expression linked to carotenoid synthesis, while XL displayed increased anthocyanin content. This is in line with the fact that the XL corolla color seems yellow-green and WT finally turns golden yellow.

### Co-analysis of DEGs and DEMs of main stages of petal color transition difference

**Figure 8:**
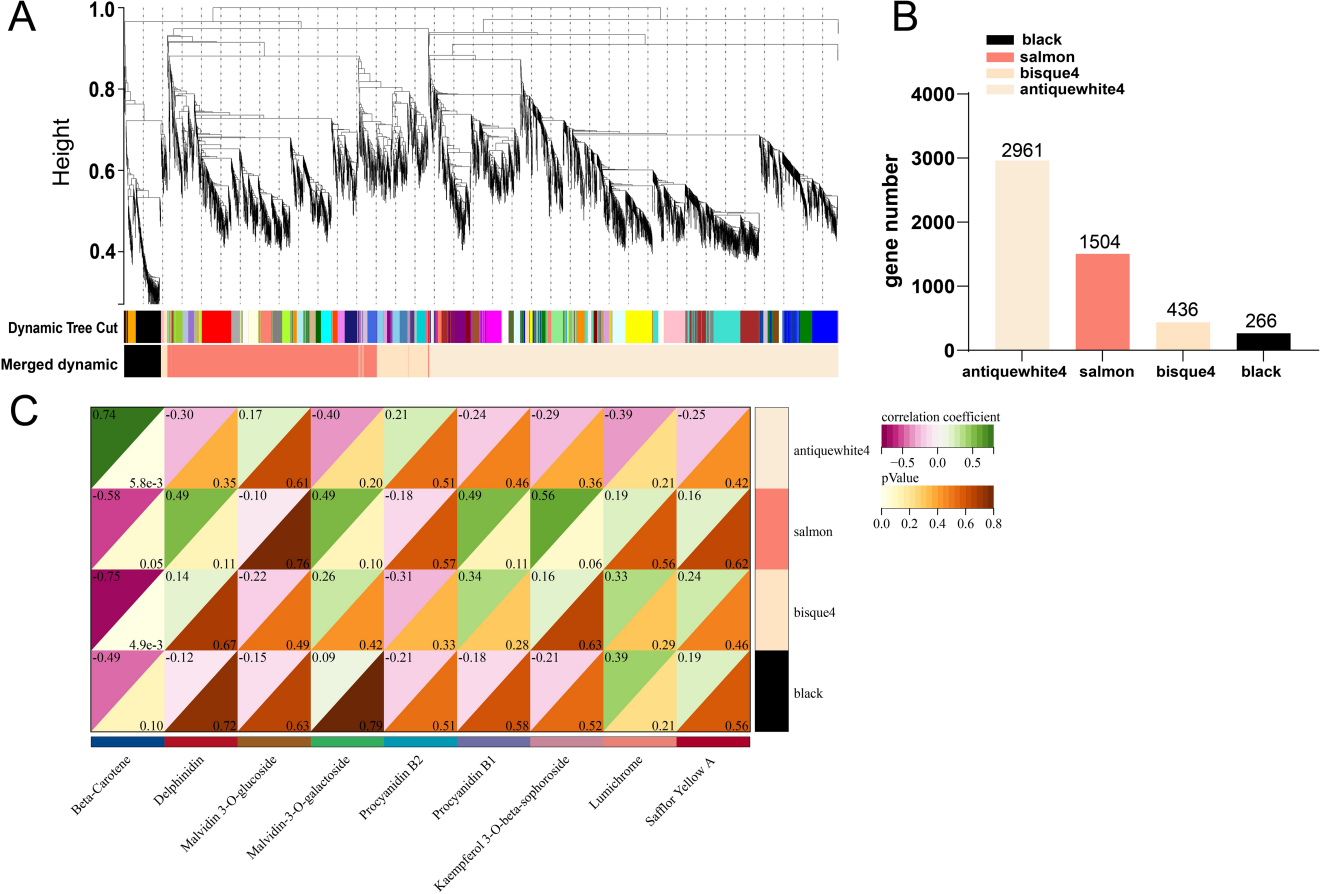
Co-expression network analysis of RNA-seq and physiological characterisation data. **(A)** clustered dendrogram of differentially expressed genes and modules identified by weighted gene co-expression network analysis. **(B)** number of genes in each module. **(C)** correlation analysis of flavonoids with gene modules.

Correlations between gene expression levels were used to build gene cluster trees (**Figure 8A**). Four common expression modules, which represent groupings of genes with substantially associated expression levels, were found in a branch of the tree (**Figure 8B**). Gene counts showed that antiquewhite4 had the most genes (2961) and Blacks had the fewest (266). The salmon and bisque4 modules had the strongest link with anthocyanin production, according to the correlation coefficients between the modules and anthocyanins and carotenoids (**Figure 8C**). There is considerable correlation between β-carotene and antiquewhite4. According to the results of KEGG annotation, carotenoid synthesis-related genes *PSY, PDS, Z-ISO, ZDS, LCYB, BCH*, and *NXS* were found to be enriched in antiquewhite4, but not in salmon or bisque4. These all point to a possible connection between the genes *PSY1, PDS, Z-ISO, ZDS, LCYB, BCH*, and *NXS* and carotenoid production, which would account for the difference in carotenoid accumulation between XL and WT.

### qRT-PCR to verify the expression of key differential genes

**Figure 9:**
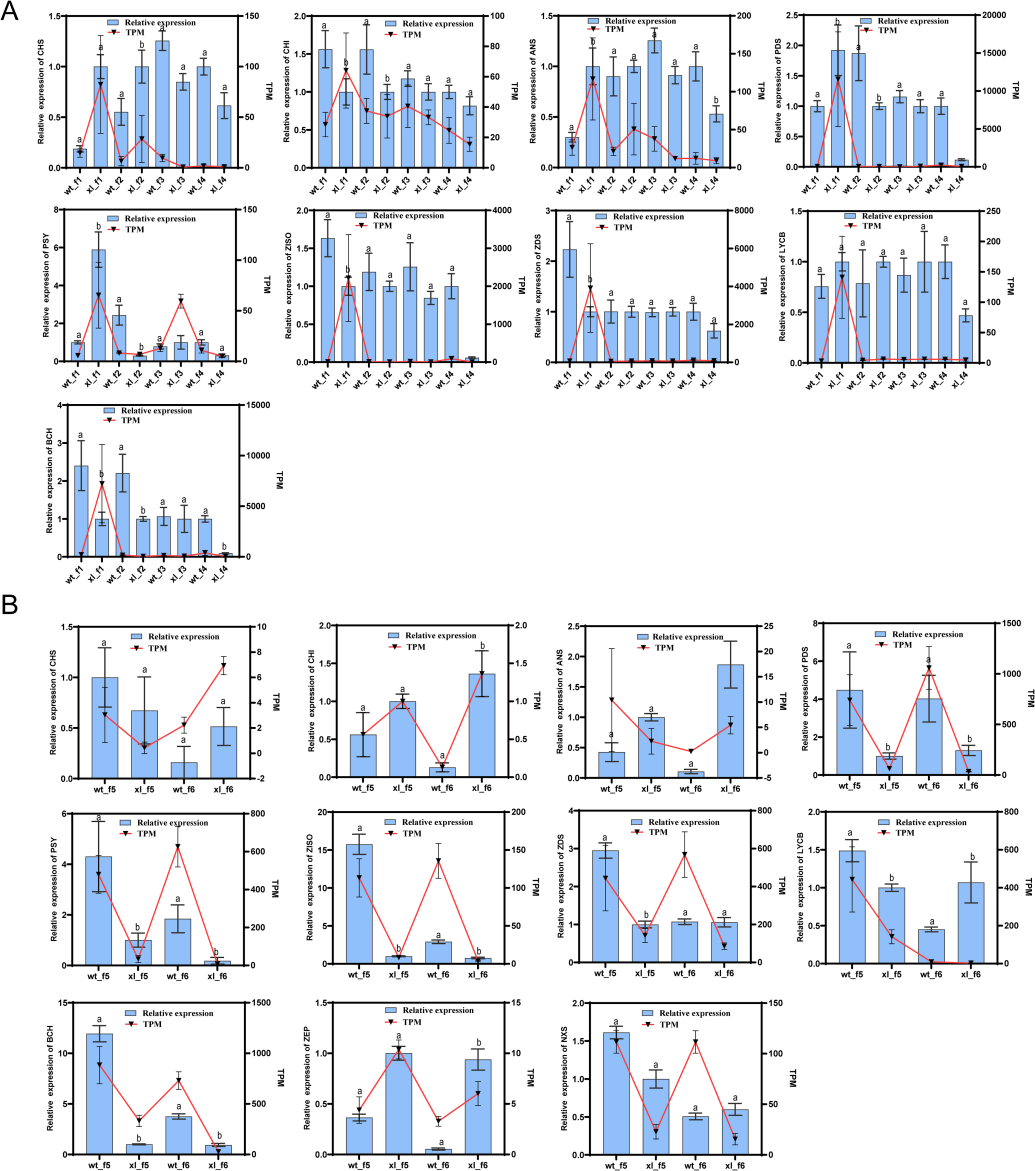
Expression of differential genes involved in anthocyanin and carotenoid synthesis was validated by qRT-PCR. **(A)** f1-f4 fluorescence quantification and transcription results. **(B)** f5-f6 fluorescence quantification and transcription results. Error bars show standard deviations, and different letters denote significant changes between sampling points:^a^ *P* > 0.05 indicates WT *vs* XL; ^b^ *P* < 0.05 indicates WT *vs* XL.

To validate the important DEG transcriptome results, qRT-PCR was used for quantitative analysis of DEGs associated with the related anthocyanin biosynthesis pathway and carotenoid biosynthesis pathway in the f1-f6 stage (**Figure 9**). The remarkable degree of agreement between these genes expression patterns and the qRT-PCR data results further supports the sequencing data dependability.

## Discussion

Transcriptome profiling and integrated metabolomics have been used more and more to study plant color synthesis (Chen et al., 2020). To the best of our knowledge, this work is the first to clarify how the color of *Lonicera macranthoide*s WT and XL is regulated. To identify the molecular foundation and metabolic processes behind the variations in flower color between XL and WT, we analyzed DEGs and DEMs associated with f1-f6 in WT and XL.

Flavonoids, chlorophylls, and carotenoids are synthesized to produce phytochromes (Chen et al., 2020). Of the many colors, the amounts of flavonoids/anthocyanins, such as paeoniflorins and common anthocyanins, determine the hues purple, blue, and red (Mizuno et al., 2021; Sunil and Shetty, 2022). The green color of photosynthetic reaction systems is due to the presence of chlorophyll (Ding et al., 2023), and carotenoids are responsible for the yellow to orange color. and yellow to orange colors are attributed to carotenoids (Mishra, 2023; Xiao et al., 2022). However, the intricate regulation of pigment production is subject to the interplay between various species, environmental factors, and their interconnections. Utilizing high-throughput sequencing, transcriptomics is the study of precisely defined and measured total RNA in an organism organs, tissues, or cells (Khozyainova et al., 2023).

Transcriptome analysis showed DEGs of 14528, 7955 and 17985 for f1-f4, f5 and f6 for WT and XL, respectively. f1-f4 was significantly enriched in flavonoids, anthocyanins, and ABC transporters (transport anthocyanin-related proteins). While the metabolic pathways of photosynthesis-tentacles, flavonoids, and carotenoid biosynthesis were highly enriched in DEGs of f5, the metabolic pathways of carotenoids and ABC transporters were primarily enriched in DEGs of f6. Next, we detected variations in transcription factor expression of enzymes linked with anthocyanin and carotenoid production in XL and WT. Anthocyanin accumulation in plants may be influenced by the expression of two important enzymes, *CHS* and *CHI*, upstream of the anthocyanin production pathway (Chia et al., 2020). The trend of anthocyanins in XL and WT at f1-f4 stage was that WT was significantly lower than XL, WT and XL tended to approach, XL was significantly lower than WT *CHS* (DN46824_c0_g4), *CHI* (DN43583_c0_g1), *ANS* (DN28844_c0_g1)), and finally XL was inverted again higher than WT, which was greenish or greenish white with both f1-f3, while WT was white at f4 stage while XL was still green, indicating that anthocyanins play an important role in color regulation at the four early stages. Two *CHS* (DN46824_c0_g6, DN46824_c0_g5) and one *CHI* (DN45113_c4_g2) were found to be significantly higher in the WT compared to the XL in f5. However, the XL in f6 did exhibit higher levels of expression for *CHS* (DN46824_c0_g6, DN46824_c0_g5), one *CHI* (DN46726_c4_g1), *ANS* (DN28844_c0_g1), and *ANS* (DN28844_c0_g1). Higher expression levels of genes *CHI* (DN46726_c4_g1) and *ANS* (DN28844_c0_g1) were observed in XL. These genes may be involved in the biosynthesis of flavonoids, such as epicatechin, chaffein, flavanone, fisetin, coriandrin, soybean flavonoids, and naringenin chalcone. *ANS*, as a flavonoid compound on the anthocyanin synthesis branch, is an important enzyme for the synthesis of colored anthocyanins from white anthocyanin substrates, causing massive pigment synthesis when it is activated (Zou et al., 2023). *ANS* expression of f6 was significantly higher in XL than WT, which may be the main reason for increased anthocyanin content (pelargonin, mallow pigment-3-O-galactoside).

In addition to being natural pigments that give fruits, vegetables, and flowers their yellow and orange hues, carotenoids help restore the flavonoid/anthocyanin content in plants when anthocyanin levels reduce (Chia et al., 2020; Liu et al., 2021). Carotenoids are involved in regulating the color of petals in flowering plants and can render petals in a variety of colors, including yellow and orange (Nisar et al., 2015). From the white to yellow petal stage, *Lonicera japonica* flower petals showed a considerable up-regulation of total carotenoids (Pu et al., 2020; X et al., 2013). *BCH* can use β-carotene to generate yellow zeaxanthin, and at the same time, the above carotenoids can also be cleaved by *CCD4* to produce large amounts of carotenoids, thus affecting plant petal color changes (Hou et al., 2016; Li et al., 2022). We found that both XL carotenoid genes were present at significantly higher levels in f1 than WT. XL was significantly downregulated in f2-f3, but not in WT. Surprisingly, WT rapidly increased *PDS* (DN55130c2g1), *ZDS* (DN54194c0g1), *BCH* (DN42921c1g2), and *CCD4* (DN50042c0g2) at f4, far exceeding XL, indicating that expression of these genes may promote yellowing of petals. Up-regulation of BCH in petals in *Dendrobium chrysotoxum* and *Ipomoea nil* may lead to increased zeaxanthin, purpura, and neoxanthin concentrations (Li and Wang, 2023; Yamamizo et al., 2010). As the corolla matured, the WT gradually changed to golden-yellow in f6, while the color of XL remained yellowish-green. XL exhibited significantly lower expression levels of the key genes upstream of carotenoid synthesis, *PSY* (DN44142_c4_g1, DN54874_c2_g1, DN49171_c1_g2), *PDS* (DN55130_c2_g1, DN34935_c0_g1), and *ZDS* (DN54194_ c0_g1), than WT at stage 5, according to the results of carotenoid differential gene expression. In the corolla of XL varieties, in f6, the levels of genes encoding *BCH* (DN42921_c1_g2), *LCYB* (DN43496_c0_g1), and *NXS* (DN34191_c0_g1) were much lower than the WT. *PSY* was found to be involved in the condensation of two geranylgeranyl diphosphate molecules to octahydrolycopene, and the up-regulation of *PSY* boosted the accumulation of carotenoids. *PSY, PDS, ZDS, LCYB*, and *VDE* are important genes in carotenoid synthesis (Fraser et al., 1994). *PDS* and *ZDS* catalyzed the desaturation of octahydro lycopene (Sun et al., 2018). Monocotyledonous plants also differ in petal color, for example mRNA levels of genes *PSY*, *PDS* and ZDS are involved in hybrid lily color change (Chia et al., 2020). *PSY, PDS, ZDS, and LYCB* could be the primary genes responsible for the variations in β-carotene production between the two types. It can be conjectured that the difference in accumulation of Carrous-like might be an important reason why WT is golden yellow while XL is yellowish green. This suggests that the difference in petals color transition between the two types of *Lonicera macranthoides* may be related to β-carotene synthesis. Furthermore, metabolomics analyses revealed that in f5, the WT accumulated much β-carotene than the XL, and in f6, the WT had the highest levels of pelargonin and mallow pigment-3-O-galactoside. This suggests that the poor accumulation of anthocyanins and carotenoids and the expression of related genes are important reasons for the difference in petal color transition between the two phenotypes of *Lonicera macranthoides.*

## Conclusion

In this study, we found that carotenoids and anthocyanins play an important role in the analysis of important candidate genes and metabolites by comparing f1-f6 *Lonicera macranthoides* DEGs and DEMs between WT and XL. Anthocyanins and carotenoids showed dynamic changes in the early f1-f4 stage, and anthocyanins and carotenoid-related gene species increased in both f5 and f6 stages, while flower color-related metabolites also increased. In summary, the difference in flower color between transition between WT and XL is related to anthocyanin and carotenoid metabolite accumulation and their related gene expression.

## Data Availability

The datasets presented in this study can be found in online repositories. The names of the repository/repositories and accession number(s) can be found below: https://www.ncbi.nlm.nih.gov/, PRJNA1040459.
