# Supplementary material for: Integrated transcriptomics and metabolomics analysis of flower petals color transition in different phenotype of Lonicera macranthoides
Source: Front Plant Sci. 2025 Jul 10;16:1605238. doi: 10.3389/fpls.2025.1605238 (PMC12287116; doi:10.3389/fpls.2025.1605238)
Supplement: Supplementary file 2 [file Image1.pdf]

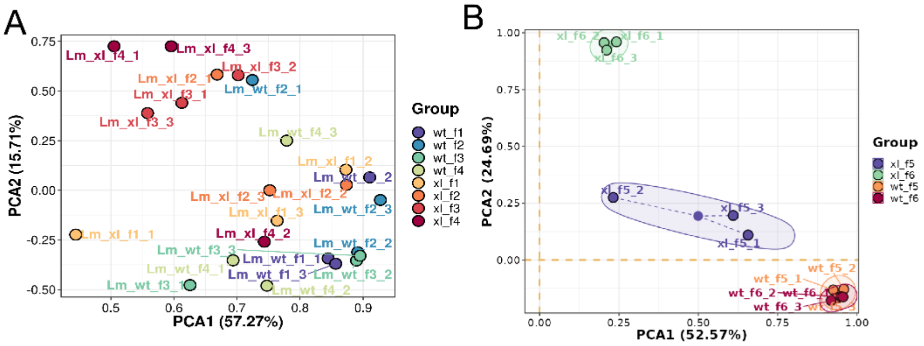

Fig. 1 Overview of Transcriptome analyses of the two varieties. (A) PCA of Transcriptome data from f1-f4. (B) PCA of Transcriptome data from f5 and f6. The first and second principal components (the rate at which differences are explained by latent variables) are represented by the coordinate axes PCA1 and PCA2 in the figure; Samples are represented by dots, and various colors denote distinct groups; Ellipses show the core regions that groups have contributed based on the 68% default confidence interval.

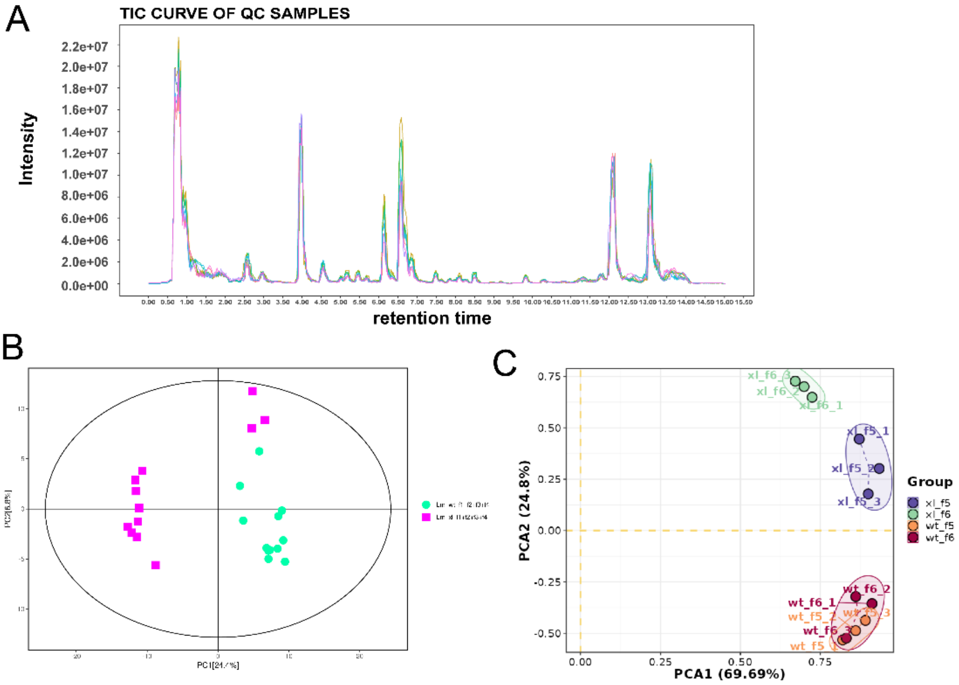

Fig. 2 Overview of metabolomics analyses of the two varieties. (A) Overlap analysis of total ion currents in different QC samples (overlap analysis of total ion currents in different QC samples indicates good instrumental stability for GC-MS analyses). (B) PCA of metabolomic data from f1-f4. (C) PCA of metabolomic data from f5 and f6. The ordinate represents the peak area, and the abscissa represents the peak retention time of QC samples. The peak retention time and peak area overlap well, indicating that the instrument has good stability. The abscissa in the figure represents the predicted principal component score of the first principal component, showing the difference between sample groups, the ordinate indicates the orthogonal principal component score, showing the difference within sample groups, each scatter represents a sample, and the scatter

shape and color indicate different experimental groups.
